# Early neurodevelopmental outcomes in children with asymptomatic congenital CMV infection

**DOI:** 10.1186/s13052-022-01387-3

**Published:** 2022-12-26

**Authors:** Maria Novelli, Fabio Natale, Anna Di Norcia, Arianna Boiani, Sara Temofonte, Francesca Calandriello, Cristina Zitarelli, Barbara Caravale

**Affiliations:** 1 Department of Human Neuroscience, Polyclinic Umberto I Hospital, Sapienza University, Via Dei Sabelli 108, 00185 Rome, Italy; 2 Department of Maternal and Child Sciences and Urology, Polyclinic Umberto I Hospital, Sapienza University, Rome, Italy; 3 Department of Developmental and Social Psychology, Sapienza University, Rome, Italy; 4 Centro NE.SVI, Rome, Italy

**Keywords:** Congenital CMV, Neurodevelopmental outcome, Neurological outcome, Bayley-III

## Abstract

**Background:**

Approximately 85–90% of congenital cytomegalovirus infections (cCMV) are asymptomatic. Few studies have investigated early and long-term neurodevelopmental outcomes in children with asymptomatic cCMV (acCMV), and the data is contradictory. In the present study, we did investigate the effect of cCMV asymptomatic infection on neurological outcomes and in cognitive, language and motor development at 6 months of age.

**Methods:**

Fifty-six children with cCMV asymptomatic infection were followed for 6 months, as part of a long-term surveillance program, examining their neurological and developmental outcomes. Neurological examination and Bayley-III Scales were performed.

**Results:**

Clinical evaluation revealed that early neurological outcomes were essentially normal, with minor neurological deficits (i.e., tone abnormalities) in a subgroup of patients. Bayley-III scores were substantially in the normal range, with 14% showing a score less than 85 (-1SD) in the Motor Scale. Children's neurological and neurodevelopmental outcomes at 6 months of age did not differ according to the trimester of infection.

**Conclusions:**

Some infants with cCMV asymptomatic infection may present minor neurological abnormalities in early stages of life. It seems useful to monitor this population for early and late neurodevelopmental sequelae.

## Background

CMV or human herpesvirus 5 (HHV-5), is a member of the family *Herpesviridae* and a human-infecting ubiquitous host–restricted virus with a world-wide seroprevalence between 45 and 100% in the adult population [1]. Currently it is known that congenital CMV (cCMV) infection can have a wide variety of clinical manifestations, ranging from asymptomatic infection to severe and life-threatening disease [2–6]. In an immunocompetent healthy host, primary CMV infection is usually asymptomatic and undetected. Sometimes it may present as a nonspecific febrile illness, or a mononucleosis-like illness characterised by fever, lymphocytosis and lymphadenopathy [7]. In immunocompromised hosts, such as human immunodeficiency virus (HIV) infected individuals or transplant recipients, the virus can result in severe and even fatal conditions [8].

Becoming infected during pregnancy may also cause problems for the foetus. Congenital cytomegalovirus (cCMV) infection is an important cause of hearing loss and neurodevelopmental delay in children; it is considered the most common infection causing newborn malformations, and it represents a significant public health problem with a prevalence of 0.2% to 2.0% of live birth pregnancies [4, 9]. Intrauterine CMV transmission may occur as a result of both primary (CMV infection in a formerly seronegative woman) and non-primary (CMV reinfection/reactivation in an already seropositive woman) maternal infection [3, 10, 11]. The percentage of cCMV infection derived from primary or non-primary maternal infections will depend on the CMV seroprevalence in the pregnant population under observation, however, countries with higher CMV seroprevalence report higher rates of cCMV infection.

cCMV infection is diagnosed by means of culture or PCR performed on saliva and/or urine up to 3 weeks of age, or, retrospectively, by detection of CMV by PCR (polymerase chain reaction) on dried blood spots (DBS) of Guthrie cards [12].

cCMV infection may be distinguished as symptomatic or asymptomatic, but there is no clear definition of the two conditions, nor definite diagnostic criteria [13].

An infected child is routinely considered asymptomatic based on the real-time PCR of saliva, urine, or both, if they have no signs of CMV disease after a clinical and instrumental evaluation [9, 14]. On the other hand, the typical definition of symptomatic infection includes the presence of multiple manifestations of the virus, such as hepatomegaly, thrombocytopenia, hyperbilirubinemia, failure to thrive, chorioretinitis, sensorineural hearing loss and/or central nervous system (CNS) involvement with microcephaly, intracerebral calcifications and ventricular dilatation [9, 15]. Approximately 11% of infants with cCMV have symptomatic infection with clinical abnormalities at birth, comprising evidence of disseminated disease and/or CNS involvement [16], and the vast majority do not show any signs of cCMV disease during the newborn period [17].

Differentiating between symptomatic and asymptomatic infection has important prognostic implications, since the former has much higher risk for adverse neurodevelopmental sequelae [17]. The negative effects of CMV on the developmental, neurologic, and audiologic functions have been clearly documented in symptomatic infants showing a high rate of intellectual disability (ID) [16, 18, 19]. Moreover, cCMV is one of the leading non-genetic causes of paediatric sensorineural hearing loss (SNHL) in developed countries, with both symptomatic and asymptomatic infections contributing to the burden of SNHL [17]. In fact, it is well known that asymptomatic infants with cCMV (acCMV) may develop SNHL throughout early childhood in nearly 10% of cases with variable onset, course, and severity [12]. However, there is still no clear data on neurodevelopmental outcomes in acCMV infected children, with few reports, often conflicting [20, 21]. There are also intrinsic methodological differences between the studies, such as sample size, the classification of symptomatic and asymptomatic infections at birth, evaluation tests, and characteristics of the population, that make these results difficult to interpret.

No significant differences were found in a large retrospective study [22] between the development of asymptomatic children with cCMV, with or without hearing impairment, and uninfected children, with a long term (18 years) follow-up when assessed using the WISC, WAIS and Bayley scales. Nonetheless, Colonna et al. [23] found that both symptomatic and asymptomatic cCMV newborns are at risk of develop long-term sequelae, particularly in the behavioural and communicative areas when assessed with NepSY, the Bells Test, BVL and TFL tests.

When considering early neurological outcomes (6–12 months), the few available studies suggest no difference in developmental indices or neurologic status when comparing acCMV children with controls using Bayley Mental Developmental Index (at 10 ± 2 months) and neurologic examination [24, 25].

Thus, the aim of our study was to analyse the early neurological and developmental characteristics of a group of acCMV infected patients through neurological assessments and Bayley-III scales. As part of a long-term surveillance program, we focused on early clinical outcomes in relation to cognitive, language, motor development and the neurologic characteristics in children with acCMV infection. We also examined which variables can affect outcomes at 6 months.

## Methods

### Participants

In the period 2013–2020 we enrolled 56 infants (30 boys, 54%; 26 girls, 46% mean age 6 months; SD 0.67) born at term (≥ 37 weeks gestational age) with acCMV infection, all delivered by women with primary CMV infection. Infants were referred to our Centre from the outpatient clinic of congenital and perinatal infectious diseases, Policlinico Umberto I Hospital of Rome, for neurological and neurodevelopmental assessments. The six-month visit is part of a larger follow-up protocol for children with acCMV, consisting of a minimum of 3 neurodevelopmental evaluations performed until 24 months of age. Further assessments (at 3 and 4 years of age) are offered to families, depending on the child's clinical status. Additionally, audiologic and ophthalmologic evaluations until school age are scheduled.

For the purpose of this study, the other inclusion criteria were: to be born full-term and have a normal audiologic evaluation in the first 6 months of age*.* Children with other diseases possibly affecting neurodevelopmental outcome (e.g., perinatal asphyxia, sepsis, chromosomal or metabolic disorders) were excluded from the study.

### Procedures

Maternal CMV primary infection was identified and dated according to serological CMV screening performed during pregnancy (IgG seroconversion or low avidity IgG and IgM antibodies) and assigned to a trimester of pregnancy. Congenital infection was established by means of viral culture (shell vial) or polymerase chain reaction (PCR) performed on urine within the first 3 weeks of life. A measure of the blood viral load (by means of PCR) was also available for all infants within the first month of life. Asymptomatic infection was established according to widely agreed procedures [26]. IUGR infants, in the absence of other signs of cCMV infection, were considered asymptomatic. Gestational age at birth, weight, and trimester of maternal infection were noted for each patient.

Written informed consent was obtained from the parents of all CMV patients for the present study, following a full explanation of the procedure(s) undertaken. The research complied with the ethical standards of the American Psychiatric Association (APA) for child development evaluation.

### Instruments

#### Neurological examination

Each participant underwent neurological evaluation, including an assessment of motor and sensory function, primitive reflexes and tone.

The neurologic examination was performed by a paediatric neurologist with long experience in the field. This was based on some fundamental items of posture, tone and movement of the Hammersmith Infant Neurological Examination (HINE) [27] and (a) asymmetries of posture (head, trunk and leg in sitting position; arms, hands, legs and feet in supine positions); (b) quality and quantity of movements and (c) passive and active tone (scarf sign, passive shoulder elevation, adductor, popliteal and ankle angles, ventral suspension and pull to sit manoeuvre) were recorded and rated as follows:

- (0) normal: no neurological abnormalities;
- (1) slightly abnormal: minimal asymmetries, minimal alteration of movements, tone and/or posture (e.g., a score of 0 or 1 on 1 or 2 items of the whole neurological assessment);
- (2) mildly abnormal: mild defects of posture, movements and/or tone not affecting function (e.g., a score of 0 or 1 on 1 or 2 items of each area of assessment);
- (3) moderately/severely abnormal: definite hypertonia or hypotonia affecting function (e.g. a score of 0 or 1 on more than 2 items of each area of assessment).

#### Bayley-III Scales

The Bayley-III Scales (BSID-III) are designed to measure the developmental functioning of young children aged 1–42 months and include Cognitive, Language, Motor Scales, administered by clinicians, and Social-Emotional and Adaptive Behavior Scales, completed by the parents [28]. For the purpose of this study, we used Cognitive, Language and Motor scales. The Cognitive Scale covers items on sensorimotor development, exploration and manipulation, object relatedness, memory, habituation, visual preference, cause-and-effect understanding, problem solving, representational and pretend play, and early learning. The Language Scale measures both receptive and expressive communication skills, assessing preverbal behaviours and communication, vocabulary use and morpho-syntactic development. The Motor Scale assesses both fine- (e.g., prehension, perceptual-motor integration, motor planning and speed, functional hand skills) and gross-motor skills (e.g., head control, stepping, standing).

Some examples of items that, according to Bayley-III Scale norms [29], a child is expected to reach at about 6 months of age are, for the Cognitive Scale: stretching out persistently for objects, manipulating a ring, retaining one block and trying to hold a second one; for the Language Scale: responding to name, interrupting activity when called by name, interacting with objects for at least 60 s, recognizing familiar words, making distinct vowel sounds and different consonant–vowel combinations, trying to get attention from others; for the Motor Scale: grasping using partial thumb opposition to the fingers, using whole hand grasp to obtain a small item, transfer objects hand to hand, sitting without a support for 30 s and rolling from back to stomach.

In this study, we computed the Cognitive, Language and Motor Composite Index Score (M = 100; SD = 15) [30]. Within each scale, the proportion of children scoring below the composite score of 85, which is considered a cut-off for distinguishing between normal development and neurodevelopmental delay, was calculated [30, 31].

#### Statistical analysis

All analyses were performed using SPSS version 25.0 for Windows (SPSS Inc. Chicago, 2017). Descriptive statistics were run and presented as percentages for qualitative variables and mean ± standard deviation for normal continuous variables. Bayley-III Scales scores were compared by trimester of infection using ANOVAs. Groups with different neurological outcomes were compared by trimester of infection using Chi square tests.

## Results

Our cohort consisted of 56 children with acCMV, 26 (46%) were males, 30 (54%) females. Clinical and demographic characteristics are reported in Table 1. Neurological examination, performed in 54/56 (96%) patients was normal (0) in 24 patients (44.4%), slightly abnormal (1) in 24 children (44.4%), and mildly abnormal (2) in 6 patients (11.1%). No children had moderately or severely abnormal neurological status (3). Tone abnormality was the most reported sign within the whole sample, with 13 patients (24%) presenting minimal or mild hypotonia.

**Table 1:** Clinical and demographic characteristics

Patients *N* = 56	
	N (%)
Timing of Maternal infection	
• 1st trimester	21 (37%)
• 2nd trimester	18 (32%)
• 3nd trimester	17 (30%)
	Mean (SD); range
Gestational age (weeks)	38.6 (1.22); 37—41
Birth weight (grams)	3182 (454); 2120—4210

Table 2 shows the neurological status of children in total, and by trimester of infection. Groups with different neurological outcomes, normal (0) vs. abnormal (1) (2), were compared by trimester of infection, and no statistically significant difference emerged between the two groups (*p* = .21).

**Table 2:** Six-months neurological examination by trimester of infection

*N* = 54				
Neurological exam	1st trimester	2nd trimester	3rd trimester	Tot
	N (%)	N (%)	N (%)	N (%)
0	11 (20.3)	5 (9.2)	8 (14.8)	24 (44.4)
1	8 (14.8)	9 (16.6)	7 (12.9)	24 (44.4)
2	1 (1.8)	4 (7.4)	1 (1.8)	6 (11.1)
3	0 (-)	0 (-)	0 (-)	0 (-)

(0) normal: no neurological abnormalities; (1) slightly abnormal: minimal asymmetries, minimal alteration of movements, tone and/or posture; (2) mildly abnormal: mild defects of posture, movements and/or tone not affecting function; (3) moderately/severely abnormal: definite hypertonia or hypotonia affecting function

Table 3 reports data from the Bayley-III Scales scores in the whole group and considering the trimester of CMV infection. The children achieved mean age-appropriate values in cognitive, language and motor areas. When considering the composite scores of each scale, 8 (14.28%) children reached scores lower than 1SD (< 85) in the Motor Scale, showing a relative motor weakness in the neurodevelopmental profile of this group. Table 3 also reports the comparison between Bayley-III Scales scores by trimester of infection through one-way ANOVA. The results showed no significant differences between the groups.

**Table 3:** Comparison between Bayley-III Scales scores by trimester of infection

*N* = 56							
	**1st trimester** **Mean (SD)**	**2st trimester** **Mean (SD)**	**3st trimester** **Mean (SD)**	**Tot** **Mean (SD)**	**Range**	***p* value**	**ANOVA**
**Cognitive (*N* = 56)**							
Composite score	106.43 (8.824)	103.33 (11.114)	102.35 (10.326)	104.20 (10.03)	85–125	n.s	F(2.55) = .87; *p* = .42;
N < .85 (%)	0	0	0	0			
**Language (*N* = 55)**							
Composite score	106.00 (8.259)	103.22 (9.861)	101.35 (10.914)	103.65 (9.68)	89–127	n.s	F(2.54) = 1.09; *p* = .34
N < .85 (%)	0	0	0	0			
**Motor (*N* = 54)**							
Composite score	95.95 (9.636)	92.33 (12.214)	93.73 (12.268)	94.13 (11.18)	73–118	n.s	F(2.53) = .51; *p* = .60
N < .85	2	3	3	8			

Normative Composite Scores as follows, M = 100, SD = 15 [31]

## Discussion

Our study aimed to evaluate children with asymptomatic congenital CMV, focusing on a preliminary in-depth analysis of developmental profiles and neurological status at an early age (6 months). To our knowledge, there are only a few studies analysing in detail the short-term outcomes of asymptomatic infected CMV children [23–25].

Neurological and neurodevelopmental assessment in our acCMV cohort revealed no major abnormalities at six months of life. It is well known that cCMV infection may cause microcephaly, periventricular calcification, ventriculomegaly or other structural abnormalities, and dysfunction of the nervous system [32]. In general, symptomatic cCMV infection is more likely to compromise neurodevelopment than acCMV infection [33] and previous studies have demonstrated that cCMV infection affects the neurodevelopment of children, with a dramatic impact on their long-term behaviours and presentations [21, 34]. Noyola et al., in 2001 [35], demonstrated that symptomatic cCMV infected children perform significantly more weakly than asymptomatic children, confirming the findings of previous research. The presence of severe motor deficits is thus well documented in children with a congenital CMV infection, while less is known about the prevalence of mild motor disorders in asymptomatic children [36]. However, only minor neurological deficits, including tone abnormalities and low scores on the motor Bayley-III scale, were found in our group.

Due to differences in design and methods, it is difficult to compare the previous literature with our results, and to draw conclusions. Many previous works found no neurological sequelae in patients with asymptomatic infection, with acCMV children performing equally well in neurodevelopmental assessments when compared with healthy controls [12]. A 10-year follow-up study showed that children with cCMV infection are unlikely to be at an increased risk of subsequent neurodevelopmental disorders if they did not show any abnormal development at 12 months of age [37]. Shan and Temple found no significant effect on psychomotor performance in acCMV infected children [25, 38]. Globally, our findings are in line with most previous studies, showing the absence of severe neurological deficits in acCMV children [24, 25].

In long-term follow-up, however, there were sporadic reports of neurologic problems in patients with acCMV infection. De Kegel et al. found that acCMV infected children did not perform significantly weaker than a control group, except on the Peabody Developmental Motor Scales-2 Gross Motor scale [36]. Zhang found that the global development quotient was lower in an acCMV group compared to controls, and that language development was usually impaired (however audiology was not reported and the effect of hearing loss on language was not analysed) [20]. Longitudinal studies with longer follow-up also suggest that an acCMV group (age 4–6 years) performed weaklier in full‐scale IQ and some motor tasks compared with the control group [38].

Thus, our preliminary results should be confirmed with further evaluations, since a negative examination for severe deficits at six months of age is not sufficient to rule out later development delays, and a long-term follow-up should be recommended.

Regarding the correlation between neurological status and timing of infection, the neurological outcome in our group of children resulted not to be associated with the trimester of infection, apparently in contrast with the literature. A first-trimester CMV maternal infection, in fact, is known to be a risk factor for neonatal symptomatic infection and possible late-onset SNHL and neurological sequelae in children. A recent study reported SNHL and neurologic sequelae exclusively in cCMVs infants following a first-trimester infection [39]. In this light, it must be noted that our group comprises only asymptomatic infections, for which neurological sequelae are rare. Interestingly, Faure-Bardon et al. (2019) reported borderline developmental delays, such as mild motor or speech delay, in roughly 9% of those children, with frequency being not significantly different according to the trimester of maternal infection.

The minor neurological abnormalities that we found in a subgroup of our patients support the hypothesis that acCMV children need prolonged and accurate follow-up programs. More studies on acCMV are needed, and longer neurodevelopmental follow-up during preschool age should be planned to confirm these preliminary findings.

## Conclusions

We provided a preliminary overview of early neurological development in a group of patients with acCMV. Our results support the idea that acCMV infection does not significantly affect neonatal neurological status or development in the early stages of life, although some children presented minor neurological signs or motor abnormalities. An active survey of acCMV children over time is recommended, in order to detect neurological disorders and consider early rehabilitation.

The strengths of the present study include the size of the selected group, with a good number of aCMV patients. The objective, as well as the inclusion and exclusion criteria, were clearly stated. Finally, the protocol and tests used are standardised.

This study also has limitations, such as the lack of a control group and a relatively brief term follow-up.

## Data Availability

The datasets used and/or analyzed during the current study are available from the corresponding author on reasonable request.

## Abbreviations

CMV: Cytomegalovirus
cCMV: Congenital cytomegalovirus
PCR: Polymerase chain reaction
DBS: Dried blood spots
acCMV: Asymptomatic cCMV
CNS: Central nervous system
